# Prospective Memory in Patients with Severe Closed-Head Injury: Role of Concurrent Activity and Encoding Instructions

**DOI:** 10.3233/ben-2009-0251

**Published:** 2010-06-29

**Authors:** Giovanni A. Carlesimo, Rita Formisano, Umberto Bivona, Lina Barba, Carlo Caltagirone

**Affiliations:** ^1^Clinica NeurologicaUniversità Tor VergataRomeItaly; ^2^I.R.C.C.S. Fondazione S. LuciaRomeItaly

**Keywords:** Prospective memory, head trauma, attention, encoding

## Abstract

*Objectives:* To assess the sensitivity of patients who suffered a severe closed-head injury to the manipulation of attentional resources and encoding instructions during the execution of prospective memory tasks.

*Material and Methods:* A group of patients with chronic sequelae of severe closed-head injury and a group of matched normal controls were given an experimental procedure for the assessment of time-based and event-based prospective memory. Availability of attentional resources at the time of intention recall and encoding conditions at the time of giving instructions were varied across experimental sessions.

*Results:* The simultaneous execution of a concurrent task was more detrimental to accuracy in the spontaneous recall of the prospective intention in the post-traumatic than in the normal control group. Moreover, the instruction to encode more extensively by rehearsing aloud and mentally imaging the actions to be performed at the time of the study improved recall accuracy more in the post-traumatic than in the normal control group.

*Conclusions:* Based on these data, we suggest that a prospective memory deficit in post-traumatic patients is due, among other things, to reduced availability of attentional resources and to poor encoding of actions to be performed.

## 1. Introduction

The term *prospective memory* (PM) refers to the ability to comply with the previously established intention to perform some action at a specified time (e.g., at five o' clock; *time-based PM task*) or when a specific event occurs (e.g., when a particular person is encountered; *event-based PM task*). As such, PM should be distinguished from the more traditionally investigated *declarative memory* which assesses the ability to remember previously occurred events or acquired information and which, in patients with organic memory disorders, is further distinguished in an anterograde and a retrograde component (memory for events occurred respectively after and before the onset of the brain disease). PM tasks share with more commonly used tasks of declarative memory the need to encode, maintaining over time and successively retrieving some new information (e.g., the list of products to be bought at the grocery). However, unlike tasks of declarative memory, in which the examiner prompts the experimental subject to initiate retrieval of the studied items at the appropriate time, in a typical PM task the subject also has to encode the association between a specific event or time and performance of the intended actions. Moreover, when the event occurs or the time expires he has to rely exclusively on his own initiative to perform the intended actions. This implies that two cognitive components are critical for the correct delayed execution of planned actions: *i*) a more typically prospective

ISSN 0953-4180/09,10/$27.50 © 2009/2010 – IOS Press and the authors. All rights reserved component, which allows reactivating the intention to perform the planned actions at the appropriate time or when the cue event occurs without any explicit external prompt, and *ii*) a retrospective memory component, which allows effectively encoding and successively recalling the cue event or time and the particular actions to be performed [17].

It is generally acknowledged that whereas the retrospective component of a PM task relies on the same declarative memory system which is also involved in the encoding and successive retrieval of past events, a variety of cognitive and motivational factors, many of which are still underspecified, contribute to the effective functioning of the prospective component [3,11, 17,20,24]. Among other things, it is debated whether and in which particular circumstances the allocation of attentional resources is a critical factor for effective compliance with a PM task. In fact, as real life PM tasks (e.g. remembering to stop at the grocery to buy something) are generally embedded in some action in progress (e.g., driving the car), it seems safe to conclude that the ability to divide attention between two simultaneous tasks is critical for effective compliance with the intended actions. Accordingly, it can be hypothesized that experimentally manipulating the attentional request of the ongoing task performed during the delay interval of a PM task should affect accuracy in the retrieval of the intended actions. Nevertheless, results of experimental studies with healthy volunteers are controversial on this issue. Indeed, although some experimental evidence document a decline of performance on the PM task as the attentional requests of the ongoing task increase [20], other data document no change of accuracy on the PM task with ongoing tasks of different complexity [23].

In the last decade, growing interest has been devoted to disturbances of PM in individuals with brain injury and neurodegenerative diseases. Indeed, difficulty in effectively complying with the execution of a delayed intention has been reported in Alzheimer's patients very early in the disease course [13], in non demented Parkinson's disease patients [8] and in patients with cerebro-vascular damage to mesio-temporal or frontal areas of the brain [29]. The assessment of PM in patients with long-term sequelae of severe closed-head injury (CHI) has consistently documented deficient spontaneous retrieval of the intended actions either when they were prompted by a cue event or were required at an established time [7,9,10,14,21]. The possible role of declarative memory deficits and reduced attentional resource availability at the origin of poor PM in these patients has been investigated by both correlational analyses and direct manipulation of the memory requests and attentional load involved in the PM task. Results of these investigations are controversial. Indeed, both significant [10,15] and non significant [21] correlation coefficients between accuracy on the PM task and performance level on declarative memory and attentional tests have been reported. Moreover, one study reported a PM deficit in patients with CHI as compared with healthy controls in a condition of high but not in a condition of low distraction [16], whereas another study documented poor performance on a PM task of a group of CHI patients irrespective of the number of cue events to be remembered (varying from one to four) [12].

Commonly used laboratory paradigms for the evaluation of PM generally fail to distinguish the relative contribution of retrospective and (properly said) prospective components to overall performance accuracy in the performance of experimental subjects. In other words, when subjects do not spontaneously initiate the intended action at the established time or at the occurrence of the cue event, these paradigms generally do not allow discriminating whether the performance failure occurred because they forgot the cue event or time and/or the specific actions to be performed or, alternatively, because they had difficulty in autonomously reactivating the prospective intention at the appropriate time despite normal remembering of the cue event or time and the specific actions. In a previous study with CHI patients carried out by our group [7], we first used an experimental paradigm specifically devised for separate assessment of the relative contribution of the retrospective and prospective memory components to the overall performance on a time-based and an event-based PM task. Briefly, subjects were instructed to perform three actions after 10 or 45 minutes had elapsed. In the time-based condition, no external event signaled expiration of the time interval; in the event-based condition, instead, the ring of a clock-timer signaled when to perform the intended actions. The spontaneous initiation of some action at the established time or at the occurrence of the cue event was a measure of the prospective component. Instead, accuracy in performing the required actions provided a measure of the retrospective component. Estimates of the prospective and retrospective components were made in a reciprocally independent way (in the sense that the performance level on one component did not influence the estimate of the performance level on the other component), because if the experimental subject did not spontaneously initiate performance of the intended actions at the established time or when the clock rang then the examiner inquired as to whether he remembered which actions he was expected to carry out. Results of that study evidenced that CHI patients performed deficiently on the prospective component of a PM task both when recall of the intention should have been prompted by expiration of the pre-established time and, to a lesser extent, when it should have been prompted by occurrence of a cue event. CHI individuals also perform deficiently in the retrospective component of a PM task; indeed, the patients in Carlesimo et al.'s study [7] were poor at reporting the specific actions to be performed also when failure to reactivate the prospective intention autonomously was circumvented by the examiner prompting them to remember the actions. Consistent with the hypothesis that declarative memory processes are mainly involved in the retrospective component of a PM task, the number of remembered actions significantly correlated with performance on a test of verbal episodic memory.

The present study was aimed at further investigating basic mechanisms of the PM impairment in patients who had suffered a severe CHI. In particular, we evaluated the hypothesis that manipulating the level of elaborative encoding at the time of task instructions or the availability of attentional resources at the time of intention recall would differently affect the performance level on the retrospective and prospective components of a PM task. The level of elaborative encoding was manipulated by giving the subjects the three actions to be performed *i*) without any encoding instruction, or *ii*) with the instruction to rehearse the actions vocally, or *iii*) with the instruction to image actually performing the actions. Instead, the availability of attentional resources was manipulated by contrasting a condition in which the time expiration (in the time-based condition) or the ring of the timer (in the event-based condition) occurred while subjects were involved in performing an ongoing task vs. a condition in which they were assigned no concurrent activity. We predicted that if, as largely documented in the literature, CHI patients' declarative memory impairment is (at least partially) due to difficulty in spontaneously engaging in deep elaborative encoding of incoming information during the study phase of a memory task [4,18,19], then requiring these subjects to rehearse or to image the action mentally would result in a larger performance improvement in the retrospective component of the PM task than that observed in healthy controls. Moreover, if the poor recall of the prospective intention in CHI patients is due to difficulty in dividing the attentional resources between two simultaneous tasks (namely, ongoing and PM tasks), then these subjects should reveal a larger improvement on the prospective component of the task than healthy participants, passing from a condition in which they are engaged in the ongoing task to a condition in which they are not performing any concurrent activity.

## 2. Methods

### 2.1. Subjects

Participants included 18 chronic survivors of severe CHI attending the Physical and Cognitive Rehabilitation Program at Santa Lucia Hospital in Rome. Eighteen normal controls (NCs), matched for age and education to patients of the CHI group, also participated in the study. The individuals with CHI had suffered a severe head trauma at least 6 months earlier (Glasgow Coma Scale at hospital admission consistently $<$ 8 [28]). All patients had recovered from a period of unconsciousness (defined as the intercurrent time between the head trauma and the first obeying of simple commands), ranging from 12 to 230 days, and were out of the following post-traumatic amnesia period. We excluded patients from the experimental sample if they had a history of alcohol or drug abuse, psychiatric or neurological symptoms preceding the head trauma, coma duration of less than 3 days, and if they were unable to attend the experimental procedures because their language, motor, or attentional deficits were too severe. The Local Ethics Committee approved the study and the patients gave their written informed consent to participate.

Personal and clinical data, as well as performance scores on some tests of the neuropsychological screening battery of the patients of the CHI group, are summarized in Table 1.

### 2.2. Experimental task

#### 2.2.1. Material

The experimental material consisted of 12 triplets of actions the patient was required to perform at the occurrence of the target event or the expiration of the established time (for example, "Switch on the light, turn off the PC and sign the roll"). Each patient was tested individually in a well-lighted room. The examiner and the patient seated opposite each other at a table on which a number of objects necessary for the execution of the prospective actions were placed. A wall clock was positioned to the left of the patient; thus, he had to turn his head to check the time.

Table 1
Demographic data (mean ± SD) of participants in the CHI and NC groups. For patients in the CHI group, some clinical index of head trauma severity and performance scores on some tests of the neuropsychological screening battery are also reported (in brackets, percentages of patients performing below the normality cut-off scores)

| | CHI ($N = 18$) | NCs ($N = 18$) |
|---|---|---|
| Sex (M/F) | 14/4 | 12/6 |
| Age (yrs) | 28.1 ± 11.2 | 27.4 ± 11.4 |
| Formal education (yrs) | 11.5 ± 2.3 | 12.5 ± 2.2 |
| Coma duration (days) | 37.9 ± 55.9 | |
| Post-traumatic amnesia duration (days) | 102.9 ± 83.3 | |
| Raven's Coloured Progressive Matrices (Carlesimo et al., 1996) | 25.9 ± 6.6 (33%) | |
| 15-word list recall (Carlesimo et al., 1996): | | |
| – immediate | 31.0 ± 13.6 (66%) | |
| –15-min delayed | 3.5 ± 4.4 (78%) | |
| Short story recall (Carlesimo et al., 2002): | 6.8 ± 4.1 (56%) | |
| Phonological word fluency (Carlesimo et al., 1996) | 21.0 ± 9.3 (72%) | |
| Modified Card sorting test (Nocentini et al., 2002): | | |
| – categories | 4.4 ± 2.1 (44%) | |
| – perseverative errors | 19.8 ± 14.3 (66%) | |

### *2.2.2. General design*

The overall experiment consisted of four sessions, each lasting about 1 hour, held over four consecutive weeks, with at least five days of interval between each one. Each experimental session included three trials of the PM task. Each trial consisted of a *study phase*, during which the examiner instructed the experimental subject about the actions he was expected to perform following 20-minutes or at the timer ring; a *delay interval*, during which he was engaged in a number of concurrent tasks that involved letters, numbers or abstract symbol barrage exercises, searching for words and orthographic errors in narrative and computer-based vigilance tests; and a *testing phase*, during which the patient's spontaneous initiative in recalling the prospective intention and his accuracy in performing the required actions were recorded.

The experimental conditions in the 12 resulting trials were varied according to three independent variables; two were alternated between experimental sessions and one within each experimental session. The first between-session variable regarded the nature of the cue prompting retrieval of the intention: in one case it was simply represented by the expiration of the 20-min. delay interval (*time-based task*), whereas in another case it was represented by the ringing of a clock timer (*event-based task*). The second between-session variable was aimed at manipulating the availability of attentional resources the experimental subject could rely on when he was expected to remember the prospective intention: in one case at the expiration of the 20-min. interval he was still involved in performing the concurrent tasks (*ongoing task condition*); in another case performance of the concurrent tasks was stopped 2 min. before the delay interval expired (*no-ongoing task condition*). Finally, the within-session variable was aimed at manipulating the quality of the encoding of the actions to be performed at the time of the study: in one case the participant simply listened to the examiner read aloud the three actions to be performed (*listening condition*); in another case the participant was required to repeat aloud the triplet of actions (*rehearsal condition*); in a third case the subject was required to image himself as he actually performed the three actions (*imaging condition*).

### *2.2.3. Procedure*

At the beginning of each trial, the examiner instructed the participant to perform three different actions after 20 min. had elapsed. The kind of instructions provided by the examiner varied depending on the encoding condition of the individual trial (see above). In the time-based experimental condition, the examiner did not add any other instructions; therefore, the expiration of the delay interval (20 min.) was not marked in any way. Although the wall clock was positioned so that it was quite visible to the patient, the examiner made no reference to the possible benefit from consulting it or a wristwatch for the purpose of time monitoring. In the event-based condition, instead, the examiner informed the patient that the end of the 20 min. would be marked by the ringing of a timer. The cue event in the event-based condition was chosen to be particularly salient (a timer ring) in order to obtain a striking contrast with the time-based condition as for the processing requests necessary to detect the event triggering the initiation of the intended actions. During the 20-min. delay interval of both the time- and event-based PM task, the subject was engaged in the above described concurrent tasks. In the ongoing task condition, the subject continued to perform the concurrent tasks until the delay interval had expired. In the no-ongoing task condition, two minutes before the time was up the examiner stopped the subject from performing the concurrent tasks. During the remaining two minutes, the subject was free from any other concurrent activity while the experimenter was involved in checking the protocols of the concurrent tasks just completed or looking in a folder for other materials. When the 20 minutes were up (in both time-based and event-based conditions), the examiner noted the actions the participant carried out spontaneously, regardless of whether or not the sequence of actions followed the order indicated by the examiner. There was a 1-min. tolerance limit, before and after the time expired, during which the individual could initiate the prospective task. If he began to perform the required actions more than 1 min. before the time expired, the examiner stopped him and said that it was not yet time. Conversely, if the patient still did not show that he remembered having to carry out some action 1 min. after the time was up, he was reminded by the examiner ("Do you remember that at this point you were supposed to do something?"). In the case of an affirmative response, the examiner recorded the number of actions carried out correctly. In this way, it was possible to obtain a separate measure of accuracy on the prospective component (autonomous retrieval of the prospective intention) and the retrospective component (remembering the specific actions to be carried out) of the PM task. Immediately after a trial was completed, the next trial began, for a total of three trials per experimental session.

Across patients, the order of the experimental sessions (time-based or event-based, ongoing task or no-ongoing task) was completely randomized. However, within the same experimental session the order of the encoding conditions (first listening, then rehearsal, finally imaging) remained fixed in order to avoid that performance of experimental subjects in the baseline condition (simply listening) was contaminated by the adoption of more effective strategies of encoding.

In summary, the experimental design included two different dependent variables: one concerned the prospective component of the experimental task (spontaneous recall of the intention to perform some action) and the other the retrospective component (accuracy in recollecting the specific actions to be performed) and three within-subject factors. We predicted that the first two factors, represented by the target event associated with the execution of the actions (time-based vs. event-based) and by the subject being engaged or not in performing some concurrent task at the time the delay interval expired, would influence the prospective but not the retrospective component of the memory task. Instead, it was expected that the third factor, represented by the quality of encoding of the actions to be performed at the time of the study, would influence the retrospective but not the prospective component of the performance.

### 2.3. *Statistical analysis*

Accuracy data distribution in both the prospective and retrospective components of the PM task was skewed to the right (especially in the NC group); therefore, the data were unsuitable for parametric statistical analysis. For this reason, non-parametric tests were consistently used for both between-subject (Mann-Whitney U test) and within-subject (Sign and Friedman tests) comparisons and correlational analyses (Spearman rank order correlation).

## 3. Results

### 3.1. *Neuropsychological screening battery*

As can be seen in Table 1, in the various tests of the neuropsychological screening battery the percentage of CHI patients that scored below the normality cut-off on tests of memory (recall of a word list and of a short story) and executive functions (phonological verbal fluency and card sorting) varied between 44% and 78%, whereas 33% of the patients performed below normal on a test of general intelligence (Raven's Matrices).

### 3.2. *Prospective memory*

Table 2 reports the percentages of accuracy for patients with CHI and for NCs in spontaneously recalling the intention to perform the required actions in the various experimental conditions.

We predicted that, consistent with previous findings by our group [7], NCs' accuracy in spontaneously recalling the prospective intention would significantly exceed that of CHI patients and that the CHI patients' deficit would be particularly severe in the time-based condition. In view of the supposed lower availability of attentional resources in head trauma patients, a critical prediction was that the advantage in the no-ongoing task with respect to the ongoing task condition would

Table 2
Proportion of accuracy in spontaneously recalling the intention to perform the required actions as a function of the different experimental conditions in the CHI and NC groups

| | Patients with CHI | | | | Normal Controls | | | |
|---|---|---|---|---|---|---|---|---|
| | Time-based | | Event-based | | Time-based | | Event-based | |
| Encoding condition | Ongoing task condition | No-ongoing task condition | Ongoing task condition | No-ongoing task condition | Ongoing task condition | No-ongoing task condition | Ongoing task condition | No-ongoing task condition |
| Listening | 0.44 | 0.44 | 0.78 | 0.67 | 0.89 | 0.78 | 1.00 | 1.00 |
| Rehearsal | 0.44 | 0.61 | 0.44 | 0.56 | 0.94 | 0.78 | 0.89 | 1.00 |
| Imaging | 0.22 | 0.61 | 0.61 | 0.72 | 1.00 | 0.83 | 1.00 | 1.00 |

Table 3
Proportion of accuracy in recalling the specific actions to be performed as a function of the different experimental conditions in the CHI and NC groups

| | Patients with CHI | | | | Normal Controls | | | |
|---|---|---|---|---|---|---|---|---|
| | Time-based | | Event-based | | Time-based | | Event-based | |
| Encoding condition | Ongoing task condition | No-ongoing task condition | Ongoing task condition | No-ongoing task condition | Ongoing task condition | No-ongoing task condition | Ongoing task condition | No-ongoing task condition |
| Listening | 0.39 | 0.44 | 0.51 | 0.54 | 0.94 | 0.90 | 0.84 | 0.87 |
| Rehearsal | 0.53 | 0.69 | 0.66 | 0.58 | 0.94 | 0.97 | 0.93 | 0.94 |
| Imaging | 0.50 | 0.55 | 0.49 | 0.64 | 0.90 | 0.89 | 0.94 | 0.92 |

be larger in the CHI than in the NC group. Finally, we did not expect any effect of the manipulation of encoding instructions at the time of the study phase on accuracy in spontaneously recalling the prospective intention.

The first prediction was supported by data analysis. Indeed, on average NCs were much more accurate than individuals with CHI in spontaneously initiating the prospective intention (0.93 *vs.* 0.55; Mann-Whitney U test: $z = 4.61$, $p < 0.001$). As expected, the encoding condition at the time of the study did not influence recall of the intention either in the group of patients with CHI (baseline 0.58 *vs.* rehearsal 0.51 *vs.* imaging 0.54; Friedman ANOVA: chi-square (2, 17) = 1.76, $p =$ n.s.) or in the NC group (baseline 0.92 *vs.* rehearsal 0.96 *vs.* imaging 0.90; Friedman ANOVA: chi-square (2, 17) = 2.94, $p =$ n.s.). For this reason, in the subsequent comparisons the accuracy level in the recall of intentions was collapsed across the encoding conditions. As for the remaining within-subjects variables, a Sign test contrasting accuracy of recall in the event- and time-based conditions documented a significant difference in the CHI group (0.63 *vs.* 0.46, respectively; $z = 2.41$, $p = 0.02$) but not in the NC group (0.98 *vs.* 0.87, respectively; $z = 1.22$, $p =$ n.s.). Finally, accuracy of recall was significantly higher when CHI patients did not perform a concurrent task than when they did (0.49 vs. 0.60, respectively; Sign test: $z = 2.41$, $p = 0.02$). However, when accuracy of recall in the ongoing and the no-ongoing task conditions were contrasted separately for the time-based and the event-based tasks, the difference turned out to be significant in the time-based (0.37 vs. 0.56, respectively; Sign test: $z = 2.41$, $p = 0.02$) but not in the event-based task (0.61 vs. 0.65, respectively; Sign test: $z = 0.60$, $p =$ n.s.). As for the subjects in the NC group, accuracy of intention recall did not differ whether they were performing the ongoing task or not when the time expired, either in the time-based (0.94 vs. 0.80, respectively; Sign test: $z = 1.22$, $p =$ n.s.) or in the event-based task (0.96 vs. 1.00, respectively; Sign test: $z = 0.71$, $p =$ n.s.). On average, accuracy in spontaneously recalling the intention passing from the ongoing to the no-ongoing task condition improved by 0.11 in the CHI group and marginally worsened by 0.06 in the NC group, which is a significant difference (Mann-Whitney U test: $z = 2.94$, $p < 0.01$).

### 3.3. Retrospective memory

Table 3 reports the number of actions correctly remembered by the patients with CHI and the NCs in the various conditions of the experimental task.

Also in this case, we expected lower accuracy in the CHI than in the NC group for the between-subject contrasts. Moreover, we predicted an effect of the encoding condition on accuracy in recalling the actions (i.e., better recall following imaging and rehearsal than just

Table 4
Correlations (Spearman Rho) between performance accuracy on the prospective and retrospective components of the experimental task and some tests of the neuropsychological screening battery

| | Progressive Matrices | 15-word list delayed recall | Short story delayed recall | Phonological verbal fluency | WCST categories | WCST perseverative errors |
|---|---|---|---|---|---|---|
| Intentions recall | 0.58* | 0.42 | 0.58* | 0.39 | 0.55* | −0.43 |
| Actions recall | 0.57* | 0.73** | 0.73** | 0.42 | 0.53* | −0.54* |

$^{*}p < 0.05$; $^{**}p < 0.01$.

listening). In particular, in view of the CHI patients' predicted difficulty in spontaneously engaging in a deep elaboration of the content of the intention, we predicted that such an advantage would be larger in the CHI than in the NC group. Instead, we did not expect that the contextual support for remembering the intention (i.e., time- vs. event-based) or the attention resources available when the delay interval expired (ongoing task vs. no-ongoing task condition) would have any effect on recollection of the specific actions to carry out.

On average, the patients in the CHI group recalled a proportion of 0.54 actions, which is remarkably lower than the control group's average (0.92) (Mann-Whitney U test: $z = 4.67$, $p < 0.001$). For the whole sample (patients with CHI and NCs), performance was not affected either by the nature of the contextual support to remember the intention or by the fact they were performing or not the ongoing task at the time the delay interval expired. Indeed, a comparable average proportion of actions was remembered in the event-based and time-based prospective tasks (0.72 *vs.* 0.74 respectively; Sign test: $z = 0.94$, $p =$ n.s.) and in the ongoing and no-ongoing task condition (0.71 *vs.* 0.74; $z = 0.00$, $p =$ n.s.). For this reason, in the following comparisons data regarding accuracy in recalling the actions were collapsed across the contextual support and the ongoing task conditions. The expected effect of the encoding conditions on accuracy in recalling the actions was reliable both in the CHI group (baseline 0.47 *vs.* rehearsal 0.63 *vs.* imaging 0.54; Friedman ANOVA: chi-square (2, 17) $= 7.18$, $p = 0.03$) and the NC group (baseline 0.89 *vs.* rehearsal 0.95 *vs.* imaging 0.92; Friedman ANOVA: chi-square (2, 17) $= 6.05$, $p = 0.04$). Post hoc analyses documented that such an effect in the CHI group was due to lower accuracy in recalling the actions in the listening than in both the rehearsal (Sign test: $z = 3.08$; $p < 0.01$) and the imaging conditions (Sign test: $z = 2.01$; $p = 0.04$), which in turn did not differ reciprocally (Sign test: $z = 1.31$; $p =$ n.s.). In the NC group, instead, only the difference between accuracy in the listening and in the rehearsal conditions was reliable (Sign test: $z = 2.25$; $p = 0.02$). Interestingly, performance accuracy passing from the baseline to the rehearsal condition increased significantly more in the CHI (0.16) than in the NC group (0.06) (Mann-Whitney U test: $z = 2.34$, $p = 0.02$). The performance improvement passing from the baseline to the imaging condition was also larger in the CHI (0.07) than in the NC group (0.03), but in this case the difference was not reliable (Mann-Whitney U test: $z = 1.63$, $p = 0.10$).

### 3.4. *Relationship between performances on the experimental task and on the tests of the neuropsychological battery*

Correlation coefficients between performance scores on the experimental task and on some tests of the neuropsychological screening battery in the CHI group are reported in Table 4. The correlation with a test of general intelligence (Raven's Progressive Matrices) and a test of executive functioning (Modified Card Sorting Test) was mostly the same for the prospective and the retrospective components of the experimental task. However, the performance on the declarative memory tests (delayed recall of a word list and of a short story) was more related to the number of actions correctly performed than to the number of intentions recalled.

## 4. Discussion

The present study was aimed at evaluating whether manipulation of the encoding instructions during the study phase or of attentional resources available at the time of the intention retrieval would affect to a different extent performance accuracy on a PM task of a group of severe CHI patients as compared with a group of matched healthy controls. Since we predicted that experimental manipulation of encoding conditions and processing resources would exert a differential effect on performance in the retrospective and prospective components of a PM task, we used an experimental paradigm specifically devised to independently assess the functionality of the two subcomponents of PM.

The participants in the present study were representative of a population of patients who had suffered a severe CHI. Indeed, they were mainly young and males and their cognitive impairment was characterized by a prevalent deficit of declarative memory and executive abilities, as expected in patients with a brain injury mostly affecting the fronto-polar and the anterior temporal cortical areas and the long-tract projection fibers in the subcortical white matter [1].

Results on the prospective component of the experimental task confirmed our prediction that the simultaneous execution of a concurrent task would be more detrimental to accuracy in the spontaneous recall of the prospective intention in the CHI than in the NC group. In fact, this expectation was confirmed in the time-based task (where the drop in accuracy in the intention recall passing from the no-ongoing task to the ongoing task condition was significantly larger in the CHI than in the NC individuals which, if somewhat, marginally increased their performance level) but not in the event-based task (where the drop in accuracy did not significantly differ among groups). Consistent with our hypothesis, we interpreted the higher sensitivity of the CHI group to the interfering effect of a concurrent activity as due to reduced availability of attentional resources, which prevented these patients from effectively managing two simultaneous cognitive tasks (i.e., the ongoing and the PM task). That such increased susceptibility to interference in the CHI patients was particularly evident in the time-based task was likely due to the larger attentional requests of this experimental condition with respect to the event-based task, particularly as the expiration of the delay interval approached. Indeed, when subjects must carry out a certain action at an established time, they have to monitor the passing of time so as not to miss the appropriate moment to perform the intended actions. As previous studies have clearly documented, the frequency of clock checks (and, therefore, the amount of attentional resources dedicated) increases strikingly in the last few minutes before the time expires [7,26]. Conversely, if the right moment is marked by the occurrence of a precise event, this will constitute a sort of "cue" facilitating recollection of the intention, i.e., an environmental support requiring less voluntary control and less attentional resource allocation than that necessary in the time-based condition [2,17,20,23]. As several studies have documented, the degree of effectiveness of the target event in eliciting the recall of the prospective intention depends on its salience, i.e., how much its perceptual characteristics stand out from the experimental context [27]. In this sense, the ringing of a clock in our experiment was highly salient, thus requiring very few attentional resources from experimental subjects in order to be detected.

A limit of the present study, possibly undermining the just reported conclusion of an attentional deficit contributing to the poor performance of CHI patients on a time-based PM task, was that we contrasted a condition in which the experimental subjects were involved in performing an attention consuming concurrent activity at the expiration of the delay interval with a condition in which two minutes before the time expiration the ongoing task was stopped and the subject was left free from any concurrent activity. This was done in order to differentiate as much as possible the two experimental conditions in terms of attentional resources absorbed by the concurrent activity. However, this manipulation might have changed the PM task into a vigilance task. This is because by definition a PM task is one that has intended actions embedded in a concurrent/ongoing task. Moreover, an unwilled bias could have been represented by the fact that the ongoing task interruption by the examiner could have acted as a reminding (a sort of anticipatory cure) for the recall of the prospective intention two minutes later. If this was the case, then the larger improvement exhibited by CHI patients with respect to the NCs passing from the ongoing to the no-ongoing task condition could be the expression of a particular deficit in these patients to manage a time-based PM task (i.e., monitoring the passing of time and autonomously self-activating at the interval expiration) which could have been, at least partially, circumvented by the availability of an external cue acting as an anticipatory reminding (in the same way as these patients were less deficitary in the event-based than in the time-based PM task). Further studies are needed to confirm that reducing the availability of attentional resources by means of a resource-consuming concurrent activity is particularly detrimental for the accuracy of CHI patients in performing a PM task. An experiment in which a group of CHI patients perform concurrent activities of different complexity (i.e., with a differential request of attentional resources) during the delay interval of a time-based PM task is currently underway in our laboratory.

A reduction of attentional resources at the base of poor PM in CHI patients was already hypothesized based on the results of previous studies. Indeed, Knight et al. [16] reported that CHI patients, as compared with matched controls, were more deficient in complying with prospective memory instructions in conditions of high- than low-distraction. Moreover, other studies reported a correlation in these patients between accuracy on a PM task and performance on tests of selective attention and executive abilities [15,25], as well as the disappearance of any significant difference between CHI participants and healthy control subjects when performance on the PM task was covaried for a measure of attention [10].

Results of the present study also confirmed the prediction that leading the experimental subjects to encode the task instructions more extensively would improve recall of the specific actions to be performed more in the CHI than in the NC group. Indeed, with respect to the no-encoding instruction condition, rehearsing aloud the triplet of actions increased subsequent performances in both CHI and NC groups. Instead, mental imaging of carrying out the actions improved performance in the CHI but not in the NC group. Moreover, the rate of improvement in the rehearsal with respect to the no-encoding condition was significantly larger in the CHI than in the NC individuals, and a similar (non significant) trend was observed for the improvement rate in the mental imagery condition. Such a pattern of results is in keeping with the hypothesis that in patients with severe CHI poor memory for the specific actions to be performed in a PM task is at least partially due to a deficit in spontaneously engaging in an effective encoding of the intended actions at the time of plan formulation. Indeed, when instructions for a deeper encoding were provided by the examiner, then the gap between NCs and CHI patients significantly decreased. The fact that such a gap was not completely eliminated could depend on the fact that the externally provided support was not strong enough to fully remediate the encoding deficit suffered by the CHI patients or, more likely, because poor recollection of the content of the intentions was not only due to encoding deficiencies but also to more structural memory problems, such as storage deficits resulting in faster forgetting of the task instructions.

The correlation analysis indirectly supports the idea that, in CHI patients, poor recall of the specific actions to be performed in a PM task could result from both declarative memory deficits and primitively extra-memory problems. Indeed, there was quite a robust association between the number of actions recalled in the PM task and the performance scores on the two declarative memory tests for verbal material. A significant relationship was also found between accuracy in action recall and performance on a test of executive functions. We interpret these findings as demonstration that in CHI patients both impoverishment of executive abilities, resulting in reduced encoding of the examiner's instructions, and a purer declarative memory deficit, leading to faster forgetting of the acquired information, concur to produce poor performance on the retrospective component of the PM task.

In a clinical perspective, our results reinforce the idea of the high prevalence and severity of the PM impairment in patients with chronic sequelae of CHI. Consistent with previous data from our own laboratory, such a deficit involves both the autonomous recall of the prospective intention at the appropriate moment and recall of the specific actions to be performed [7]. The novelty of the data presented here is that they suggest a reduced availability of attentional resources at the base of poor recollection of the prospective intention and an impoverished ability to encode the prospective instructions at the base of the poor recollection of the specific actions to be performed. Such a functional analysis of the cognitive deficits underlying poor PM in CHI patients could be of help in planning sound rehabilitation interventions aimed at reducing the debilitating impact of PM deficits in these individuals. In particular, cognitive training aimed at improving deficits of attention (particularly during execution of the dual tasks) could have a positive impact on CHI individuals' compliance with delayed intentions.

## Acknowledgments

We are profoundly indebted to Paola Casadio who made an invaluable contribution to the planning of the present study and to the data collection until the time of her departure.

This research was supported by a grant from the Italian Minister for University and Research – COFIN 2005.
